# A Child Lost to Follow Up Carrying Beta Thalassemia Major: A Case Report

**DOI:** 10.31729/jnma.5129

**Published:** 2020-06

**Authors:** Prakash Banjade, Jeetendra Bhandari

**Affiliations:** 1 Department of Internal Medicine, Gan Regional Hospital, Maavah Health Center, Republic of Maldives; 2 Department of Emergency Medicine, Chitwan Medical College, Chitwan, Nepal

**Keywords:** *Beta Thalassemia*, *case report*, *follow up*, *Maldives*

## Abstract

Thalassemia is inherited autosomal recessive disorders characterized by reduced rate of hemoglobin synthesis due to a defect in alpha or beta globin chain synthesis. Maldives has a beta thalassemia prevalence rate of 16-18%. Classical symptoms of beta thalassemia are common on those patients who present late for blood transfusion which is common among the south Asian countries due to resource poor situation. This case is a rare case report of commonly occurring phenomenon which has been reported less among south Asian region. Reporting this case will help health worker to manage cases accordingly. A five and half year prior diagnosed case of beta thalassemia at age of 2 years and lost to follow up presented with cough, Dyspnoea, Irritability, fatigue with classic symptom of beta thalassemia. She was managed with blood transfusion and kept on continuous follow up for transfusion and iron overload management.

## INTRODUCTION

Thalassemia is inherited autosomal recessive disorders characterized by reduced rate of hemoglobin synthesis due to a defect in alpha or beta globin chain synthesis.^1^ It has high cases in Mediterranean, Middle east, Central Asia, Indian Subcontinent and Far East.^2^ Maldives has a beta thalassemia prevalence rate of 1618 %.^3^ Major reason for high cases in these regions is due to high pressure of Plasmodium falciparum malaria infection.^4^ Beta Thalassemia majorpresents as severe anaemia, Skeletal anomaly with typical clinical and radiological manifestations.^5^ More classical symptom are observed among the non or under treated patients.^2^

## CASE REPORT

A five and half year-old girl, from Cozy corner, L Maavah, Maldives presented to Emergency unit. She presented with cough, Dyspnea, Irritability, and fatigue. She had no fever. She was diagnosed case of Beta Thalassemia. She was diagnosed at the age of 2 years. She was diagnosed and lost to follow up. Her family history was not significant for any blood related disorder or any genetic disease.

**Figure 1:**
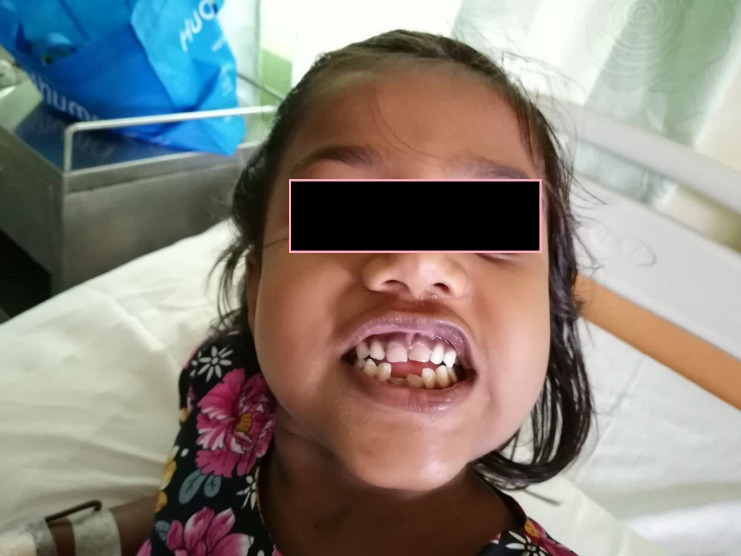
Chipmunk face with dental anomaly of a child diagnosed with beta thalassemia failed to follow up.

On physical examination patient was ill looking. Her vitals were stable. She was clinically anemic with brittle hair and nail. Patient’s finger nails and skin extremities exhibited whitish tinge and sclera showed pallor. Her skin was ashen grey in color. She appeared dehydrated and had a body weight of 13.11 kg. She was underbuilt, under-nourished with a short stature, with evident icterus, and yellow tinged fingernails. Decayed upper tooth, not associated with pain or swelling. Head and Neck examination showed maxillary expansion, retracted upper lip and saddle nose; all together depicting the classical “Chipmunk facies”. Also noted yellowish tinge at the junction of hard and soft palate. Intraoral examination, localized periodontitis and broken teeth in lower anterior aspect as shown (Figure 1). Her abdominal examination didn’t show and sign of enlargement of spleen. Her ophthalmologic and audiologic examination were done and were within normal limits.

Haematological examination was performed. Her haemoglobin was 4.5 gm/dl. Hematologic investigation revealed microcytic hypochromic anaemia with anisocy-tosis, poikilocytosis, nucleated Red Blood Cells (RBC). The impression drawn from the peripheral smear study was that of haemolytic anaemia favouring Thalassemia going for haemolytic crisis. Later Haemoglobin (Hb) electrophoresis was done which too was in favor of Beta Thalassemia major. Her Human immunodeficiency Virus (HIV), Hepatitis B, and Hepatitis C was negative. Liver function test and Renal function test were within normal limit.

She was planned for blood transfusion. She was given 3 pints of packed cell. She was given each packed cell at the rate of 220 ml in every 4 hours. Her vitals were monitored regularly during the transfusion to see any transfusion related complications. No complication ware obtained during transfusion. Then she was investigated for Haemoglobin (Hb) and found out to be 9.5 gm/dl. She was then discharged and advised to follow up in 15 days. In follow up visit her ferritin was investigated and was found to be 3562.69 ng/ml. Then she was started on iron chelating agent. She was kept on Deferoxamine B(DFO) 2 gm per dose at the rate of 4 times in a week and oral Deferasirox 400 mg once in a day dose. Her Hb was 7.5 mg/dl, she was transfused with a pint of packed cell and discharged hose with a follow up in 20 days for transfusion. She is hospitalized every 20 days for transfusion of packed RBC. She is investigated for Liver function test and thyroid function testis every 20 days. Her growth and development is assessed in every OPD visit for follow up.

## DISCUSSION

Individual with beta thalassemia usually present in early life within first 2 years of life which was similar in this case where she was presented at the age of 2 years.^6^ Patient presenting with beta thalassemia need regular blood transfusion from the early age. If patient miss to have blood transfusion then the classic clinical picture appears on patient. Classical clinical picture includes growth retardation, pallor, jaundice, brown pigmentation of skin, poor musculature, genu varum, hepatosplenomegaly, leg ulcers, development of masses from extramedullary haematopoiesis and skeletal changes that is due to bone marrow expansion. The changes in skeleton including deformity of long bones of the legs and typical craniofacial changes including bossing of the skull, prominent malar eminence, depression or bridges of nose, tendency to a mongoloid slant of the eye and hypertrophy of maxillae, which tends to expose the teeth.^2^ In the child that presented has some of the feature like these which might be due to the late starting of blood transfusion due to lost to follow up. The classic symptoms like these are only seen in developing countries, in which the resources for carrying out long term transfusion program are not available or patient tend to care less for the disease.^2^ If regular transfusion of blood is started early and continued then growth and development occurs for first 10-12 years.^7^ In this case the development has been hampered due to lost to transfuse the blood.

Patient with Thalassemia major have a severe microcytic and hypochromic anaemia. Peripheral blood smear shows anisocytosis, poikilocytosis (speculated tear drops and elongated cells) and nucleated RBC.^6^ Most of features were seen in our case too suggesting the classic case of beta thalassemia. The treatment modality is always blood transfusion in a regular basis. Transfusion was performed to this patient too. Regular transfusion corrects anaemia, suppress erythropoiesis and inhibit increased gastrointestinal absorption of iron.^6^ According to Thalassemia International Federation transfusion has to be done every 2-3 weeks^8^ which was performed in our case too.

The most common secondary complication that is due to transfusion is iron overload. Which can be assessed through the level of ferritin level. Complication can be overcome by the use of chelating drugs like DFO and Deferasirox.^6^ Despite of chelating agent one can have growth retardation and failure of sexual maturation. Complications of iron overload adults with Human homeostatic iron regulator protein (HFE)-associated hereditary hemochromatosis: involvement of heart (dilated cardiomyopathy and pericarditis), liver(chronic hepatitis, fibrosis, and cirrhosis) and endocrine glands( diabetes mellitus, hypoparathyroidism, hypothyroidism, hypopituitarism and low adrenal secretion).^6^ For the complication of iron overload serum ferritin has to be investigated along with endocrine function, cardiac function.^6^ Regular blood monitoring for the above condition that may appear has been performing in our patient too. Along with the blood work the semi-annual assessment of growth and development has to be done for every paediatric patient.^6^

Although a common disease among south Asian Population, case reports are very less reported on different circumstance. We have tried to report case that has failed to follow up despite the diagnosis. Complication can occur in any patient who deny to follow up so proper counselling should be done so that the patient comes in regular follow up and save the patient that can be prevented with timely intervention. Along with this the community should take active participation to help the patients for regular follow up and proper supply of blood needed for the patient.
